# Moxibustion as an Adjuvant Therapy for Chronic Kidney Disease: A Systematic Review and Meta-Analysis of 23 Randomized Controlled Trials

**DOI:** 10.1155/2020/6128673

**Published:** 2020-10-28

**Authors:** Xu Zhou, Qingni Wu, Yanping Wang, Qing Ren, Weifeng Zhu, Ziqian Yao, Jianrong Chen

**Affiliations:** ^1^Evidence-Based Medicine Research Center, Jiangxi University of Traditional Chinese Medicine, Nanchang, Jiangxi, China; ^2^Department of Orthopedics and Traumatology, Li Ka Shing Faculty of Medicine, The University of Hong Kong, Hong Kong SAR, China; ^3^Department of Endocrinology, The First Affiliated Hospital of Nanchang University, Nanchang, Jiangxi, China

## Abstract

**Objective:**

This systematic review aims to investigate the efficacy and safety of moxibustion for chronic kidney disease (CKD).

**Methods:**

Nine databases were searched to identify relevant evidence up to March 8, 2020. Randomized controlled trials (RCTs) that tested moxibustion + basic treatments versus basic treatments alone for patients with CKD and reported, at least, one of the outcomes of interest were included. In the meta-analyses, the mean differences (MDs) and 95% confidence intervals (CIs) were used to measure the effect size.

**Results:**

Twenty-three RCTs (*n* = 1571) with a moderate to high risk of bias were included. The pooled estimates showed that compared with the controls, patients after moxibustion had a significant reduction in serum creatinine (MD −17.34 *μ*mol/L, 95% CI −28.44 to −6.23; *I*^2^ = 87%), 24-hour urine protein excretion (MD −0.75 g/h, 95% CI −1.07 to −0.42; *I*^2^ = 84%), and blood urea nitrogen (MD −0.63 mmol/L, 95% CI −1.09 to −0.18; *I*^2^ = 37%) and a significant improvement in the quality of life (MD 10.18, 95% CI 3.67 to 16.69; *I*^2^ = 57%). Moxibustion did not show a significant effect on the estimated glomerular filtration rate (eGFR), creatinine clearance, or hemoglobin. The subgroup analyses showed that a longer course of moxibustion (>8 weeks) and indirect moxibustion had a greater effect on reducing serum creatinine. The effect of moxibustion on blood urea nitrogen changed to be nonsignificant after excluding RCTs with a high risk of bias (MD −0.96 mmol/L, 95% CI −2.96 to 1.03). Only one adverse event of burn was reported.

**Conclusions:**

This systematic review suggests that, as an adjuvant therapy, moxibustion may improve serum creatinine, urinary protein excretion, blood urea nitrogen, and quality of life in patients with CKD. Moxibustion may not have effects on eGFR, creatinine clearance, or hemoglobin. The quality of evidence is weakened by the limitations of risk of bias, heterogeneity, and imprecision.

## 1. Introduction

Chronic kidney disease (CKD) is a kind of disease characterized by the progressive impairment of renal function [1]. One-third of patients with CKD will ultimately progress to end-stage kidney disease over ten years and have to receive dialysis or kidney transplantation, resulting in a reduction in quality of life and shortening of life expectancy [2]. CKD is becoming a major global health problem with a 1.028-fold increasing annual incidence (15.0 million cases in 2007–19.7 million cases in 2017) [3]. In 2016, the global prevalence of CKD was up to approximately 12% (75.27 million), leading to more than 1.19 million of patients deaths [4, 5].

Although the causes vary (e.g., primary glomerulonephritis, diabetes, systemic lupus erythematosus, and hypertension, gout), patients with different types of CKD have common pathological damage, including nephron loss, nephron hypertrophy, impaired glomerular filtration, and renal fibrosis and often reach similar outcomes [6]. In the current guidelines of Western medicine, the main management for CKD is to control the primary diseases and treat complications (e.g., anemia, malnutrition, and mineral and bone disorders), which could delay the progression of CKD and improve the quality of life to some extent [7–9]. The progression of CKD is hard to be prevented by these treatments [10]. Today, researchers are still exploring new approaches that could improve the outcomes of CKD.

In China, acupoint stimulation therapies represented by acupuncture have been attempted in patients with CKD, and there has been a small amount of evidence for its efficacy [11]. However, because of the limitation of invasiveness, acupuncture seems to induce a high risk of infection in patients with CKD whose immunity is often impaired [12]. The clinicians, therefore, turned their attention to moxibustion, a local thermotherapy on acupoints that burns moxa cones or sticks, to generate a warming stimulation [13]. Moxibustion provides a safer therapeutic effect than acupuncture by comprehensively delivering the medicinal effect of moxa, thermal and light radiation effect of burning of moxa, and aromatherapeutic effect of the combustion products of moxa [14]. To date, clinical studies have shown that moxibustion is effective in several primary diseases of CKD, such as diabetes, hypertension, and rheumatoid arthritis [15–17]. Some studies have also suggested that moxibustion can improve osteoarthropathy and asthma, which represents its anti-inflammatory and immunomodulatory effects that are probably linked to the treatment of CKD [18, 19]. In addition, an experiment of a CKD animal model has showed that after 12 weeks of moxibustion at Shenshu (BL-23) and Geshu (BL-17), the levels of serum creatinine and urea nitrogen decreased significantly [20].

Therefore, we hypothesized that moxibustion could be an effective and safe complementary intervention for CKD and performed a systematic review aiming to assess and synthesize all currently available randomized controlled trial (RCT) evidence on moxibustion for treating CKD.

## 2. Materials and Methods

We followed a predesigned protocol registered on the PROSPERO platform (No. CRD42019131809). The reporting of this review was guided by the Preferred Reporting Items for Systematic Reviews and Meta-Analyses (PRISMA) statement [21].

### 2.1. Literature Search

We searched four English databases (PubMed, EMBASE, the Cochrane Library, and Clinicaltrials.gov) and five Chinese databases (SinoMed, Chinese National Knowledge Infrastructure, WanfangData, VIP, and Chinese Clinical Trial Registry) to identify the literature related to moxibustion for CKD up to March 8, 2020. No restrictions on language or publication status were imposed in the search. The detailed search strategies in each database are compiled in Table S1 in the supplementary files. We also looked through the references of relevant reviews to identify other possible studies for inclusion.

### 2.2. Eligible Criteria

An eligible study had to be a parallel or crossover RCT that tested moxibustion combined with basic treatments versus basic treatments alone for patients with CKD and reported, at least, one of the outcomes of interest. The diagnosis of CKD should be based on an official standard, such as the Kidney Disease Outcome Quality Initiative (K/DOQI) standard [22]. RCTs enrolling patients undergoing kidney transplantation were excluded. Any type of moxibustion that burned moxa materials was eligible, except for invasive blistering moxibustion. Moxibustion-like thermotherapy that did not involve the burning of moxa was ineligible, such as electromoxibustion and infrared laser moxibustion. The eligible basic treatments included the treatments for the primary diseases, the correction of electrolyte disorders and anemia, nutritional support, and dialysis. The combination of any other acupoint stimulation therapies, such as acupuncture, acupressure, and catgut embedding, was not allowed in either group.

### 2.3. Outcomes

The primary outcomes were defined as changes in serum creatinine (*μ*mol/L) and the estimated glomerular filtration rate (eGFR) (mL/min/1.73 m^2^). The secondary outcome included changes in 24 hour urine protein excretion (g/h), creatinine clearance (mL/min), hemoglobin (g/L), blood urea nitrogen (mmol/L), and quality of life assessed by any validated scale.

### 2.4. Literature Screening and Data Extraction

Two reviewers, independently and repeatedly, screened the literature and extracted data. The reviewers first excluded the irrelevant literature by reading titles and abstracts and finally determined the eligibility by checking the full text of the articles. The data extracted from the included RCTs were as follows: author, publication date, sample size, patients' sex and age, stage and classification of CKD, type, acupoint, frequency and course of moxibustion, length of follow-up, and outcome data. If there were multiple follow-up data for the same study population, only the last follow-up data were retained. For the crossover trials, only the first-stage data were utilized. The reviewers cross checked the extracted results and resolved inconsistencies by discussion or seeking the help of a third reviewer.

### 2.5. Risk of Bias Assessment

The risk of bias assessment was based on Guyatt's revision of the Cochrane risk of bias assessment tool [23]. It assesses selection bias through two items “whether the random sequence generation method is appropriate” and “whether the allocation concealment method is appropriate,” performance bias through one item “whether the patients and clinicians correctly are correctly blinded,” detection bias through one item “whether the outcome evaluators are correctly blinded,” detection bias through one item “whether the results data was complete,” reporting bias through one item “whether the RCT is free from selective reporting,” and other biases through one item “whether there are other sources of bias.” The reviewers first rated each item as definitely low, definitely high, or uncertain risk and, then, inferred the uncertain items to be probably low risk or probably high risk from the relevant information throughout the article. Finally, the reviewers classified the overall risk of bias risk for each RCT: (1) low: all items were low risk; (2) high: at least one definitely high-risk item or more than 4 probably high-risk items; and (3) moderate: the rest. The risk of bias was assessed by two reviewers, the process of which was independent and repeated. Any disagreements were addressed by discussion or seeking help from a third reviewer.

### 2.6. Statistical Analysis

We performed meta-analyses using a random-effects model based on the DerSimonian and Laird estimation [24]. The data type of all outcomes was continuous, so the effects were measured by the mean differences (MDs) and 95% confidence intervals (CIs). Data from individual RCTs were pooled by the inverse variance method. Heterogeneity across studies was tested by using the chi-squared test and I^2^ statistic where a *P* value of <0.10 in the chi-squared tests or I^2^ ≥ 50% indicated statistically significant heterogeneity [25].

According to the guidance proposed by Sun et al. [26], we performed four preset subgroup analyses to explore the source of heterogeneity:

1. Patients with mild-to-moderate CKD were expected to have a better efficacy than those with severe CKD; the mild-to-moderate CKD was defined as CKD stage 1–3, eGFR ≥ 30 mL/min/1.73 m^2^, or <442 *μ*mol/L, and the severe CKD was defined as CKD stage 4-5, eGFR < 30 mL/min/1.73 m^2^, >442 *μ*mol/L, or undergoing dialysis [22, 27, 28].
2. Indirect moxibustion was expected to have better efficacy than direct moxibustion. Direct moxibustion was defined as moxibustion where the igniting moxa was placed on top of the acupoints, and indirect moxibustion included ginger-separated moxibustion, aconite cake-separated moxibustion, long-snake moxibustion, and herbal cake-separated moxibustion.
3. A longer course of moxibustion (>8 weeks) was expected to have better efficacy that a shorter course of moxibustion (≤8 weeks).

To validate the robustness of the meta-analytic results, we also performed sensitivity analyses excluding RCTs with a high risk of bias. For the outcomes with a number of studies ≥10, we drew funnel plots and conducted Egger's regression tests to detect publication bias [29]. The statistical software used was the “meta” package for R version 3.6.2 (Ross Ihaka, Robert Gentlemen, New Zealand).

## 3. Results

### 3.1. Results of the Search and Screening

**Figure 1:**
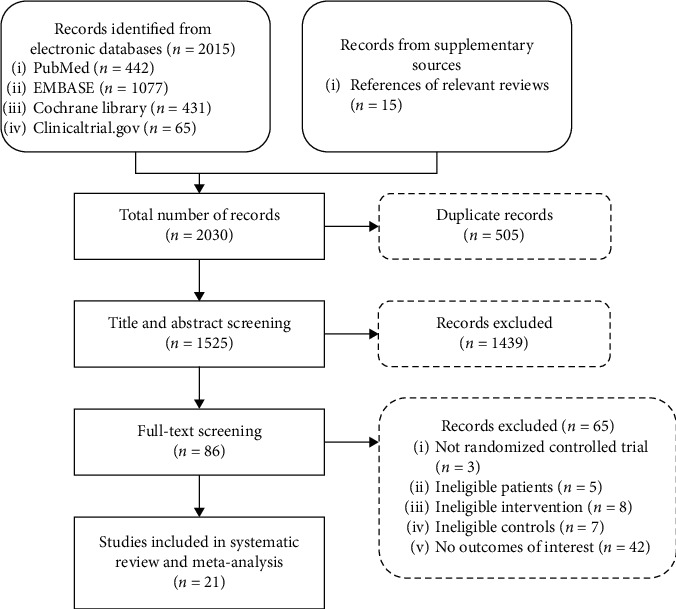
Flowchart of study screening.

We identified 2004 records from the initial search and finally included 23 RCTs [30–52] that enrolled a total of 1571 patients. The search and screening process is shown in Figure 1.

### 3.2. Characteristics of the Included Studies

Table 1 summarizes the basic characteristics of each RCT. The included RCTs were small in sample size (15 to 60). Fifty-one percent of patients were male, and the patients' average age ranged from 22.4 to 69.7 years. In individual studies, there was no significant difference in the important confounders, such as age, sex, or course of disease, between the moxibustion group and the control group. Thirteen RCTs tested direct moxibustion [31, 32, 34–40, 42, 46, 48, 49] and 12 tested indirect moxibustion [30, 33, 41, 43, 45, 47, 50–52]; the acupoints most commonly used were ST36 [34, 36–40, 43–48, 51, 52] (14 RCTs), BL23 [36, 38, 40, 43–47, 51, 52] (10 RCTs), and BL20 [36, 40, 43, 44, 46, 48, 51, 52], CV04 [31, 32, 35, 40, 42, 49, 51, 52], SP06 [34–37, 39, 43, 45, 46], and GV04 [40, 44–47, 50–52] (8 RCTs each). The course of moxibustion treatment was ≤8 weeks in 16 RCTs [30–36, 39–43, 45, 47, 49, 50] and >8 weeks in 7 RCTs [37, 38, 44, 46, 48, 51, 52].

### 3.3. Results of the Risk of Bias Assessment

**Figure 2:**
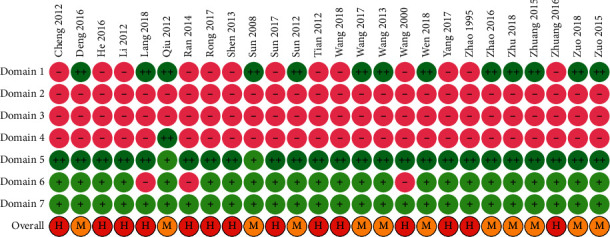
Risk of bias assessment: notes: Domain 1: random number generation; Domain 2: allocation concealment; Domain 3: blinding of patients and clinicians; Domain 4: blinding of outcome evaluators; Domain 5: completeness of outcome data; Domain 6: selective reporting; Domain 7: other sources of bias; “++”: definitely low risk of bias; “+”: probably low risk of bias; “−“: probably high risk of bias; H: overall high risk of bias; and M: overall moderate risk of bias.

A random number table was used to generate the randomization sequence in 12 RCTs. None of the RCTs mentioned whether they implemented allocation concealment. Only one RCT reported that the outcome evaluators were blinded. All studies had complete follow-up. Two RCTs [33, 44] were suspected to selectively report the outcomes because of the lack of reporting important outcomes. Overall, 12 (52.2%) studies [30, 32, 33, 35, 36, 38, 40, 41, 44, 46, 47, 50] were rated as having a high risk of bias, and 11 (47.8%) studies [31, 34, 37, 39, 42, 43, 45, 48, 49, 51, 52] had a moderate risk of bias (Figure 2).

### 3.4. Serum Creatinine

**Figure 3:**
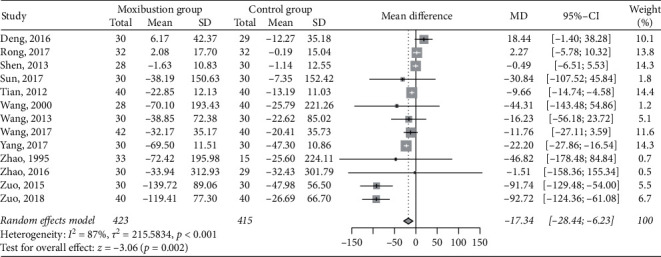
Meta-analysis of moxibustion versus nonmoxibustion treatment for serum creatinine (*μ*mol/L).

Thirteen RCTs [31, 35, 36, 38, 40, 42–44, 46–48, 51, 52], including 423 patients in the moxibustion group and 415 in the control group, reported data on serum creatinine before and after treatment. As shown in Figure 3, patients receiving moxibustion had a reduced level of serum creatinine to a greater extent compared with those receiving basic treatments alone (MD −17.34 *μ*mol/L, 95% CI −28.44 to −6.23, *P*=0.002). The heterogeneity across the included studies was high (I^2^ = 87%).

### 3.5. Estimated Glomerular Filtration Rate

**Figure 4:**
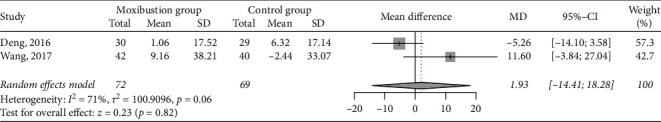
Meta-analysis of moxibustion versus nonmoxibustion treatment for eGFR (mL/min/1.73 m^2^).

There were only 2 RCTs [31, 42] with 141 patients testing changes in eGFR. Compared with the control, the moxibustion treatment had no obvious advantages in improving eGFR in the treatment of CKD (MD 1.93 mL/min/1.73 m^2^, 95% CI -14.41 to 18.28, *P*=0.82; I^2^ = 71%; Figure 4).

### 3.6. 24 Hour Urine Protein Excretion

**Figure 5:**
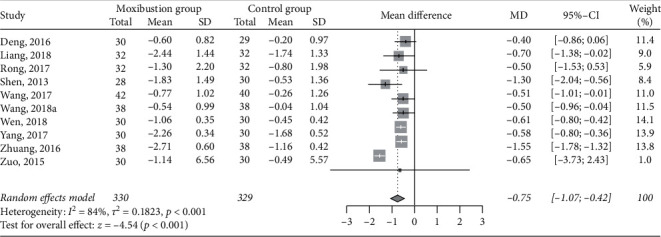
Meta-analysis of moxibustion versus nonmoxibustion treatment for 24 hour urine protein excretion (g/h).

Ten RCTs [31, 33, 35, 36, 41, 42, 45, 46, 50, 52] reported data on changes in 24 hour urine protein excretion. There were a total of 330 patients in the moxibustion group and 329 patients in the control group. The pooled effects favored the moxibustion group in reducing 24 hour urine protein excretion (MD −0.75 g/h, 95% CI −1.07 to −0.42, *P* < 0.001; Figure 5), with high study-level heterogeneity (I^2^ = 84%).

### 3.7. Creatinine Clearance

**Figure 6:**
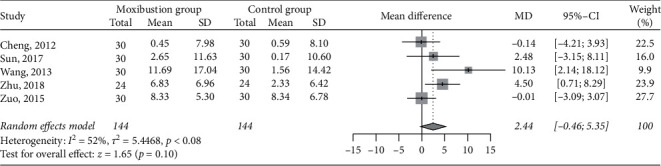
Meta-analysis of moxibustion versus nonmoxibustion treatment for creatinine clearance (mL/min).

Five RCTs [30, 38, 43, 49, 52] had data on changes in creatinine clearance before and after treatment, involving 144 patients in each group. The results of the meta-analysis did not show a significant difference between the moxibustion group and the control group in the increase in creatinine clearance after the treatments (MD 2.44 mL/min, 95% CI −0.46 to 5.35, *P*=0.10; Figure 6). The heterogeneity was moderate (I^2^ = 52%).

### 3.8. Blood Urea Nitrogen

**Figure 7:**
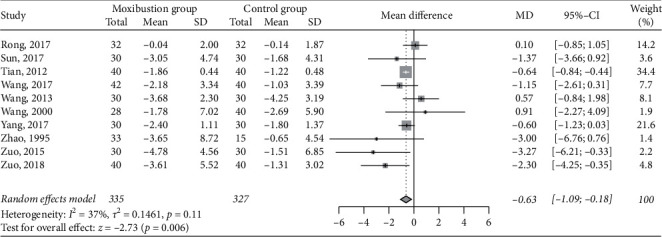
Meta-analysis of moxibustion versus nonmoxibustion treatment for blood urea nitrogen (mmol/L).

Compared with nonmoxibustion treatment, moxibustion significantly reduced the level of blood urea nitrogen (MD -0.63 mmol/L, 95% CI −1.09 to −0.18, *P*=0.006; Figure 7), which was supported by pooling data from 10 RCTs (*n* = 662) [35, 38, 40, 42–44, 46, 47, 51, 52]. The heterogeneity was low (I^2^ = 37%).

### 3.9. Hemoglobin

**Figure 8:**
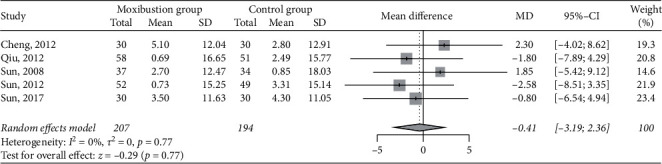
Meta-analysis of moxibustion versus nonmoxibustion treatment for hemoglobin (g/L).

Five RCTs (*n* = 401) [30, 34, 37–39] involving 207 and 194 patients in the moxibustion and control groups, respectively, compared data on hemoglobin levels. The pooled result did not favor any group regarding changes in hemoglobin after the treatments (MD −0.41 g/L, 95% CI −3.19 to 2.36, *P*=0.77; I^2^ = 0%; Figure 8).

### 3.10. Quality of Life

**Figure 9:**
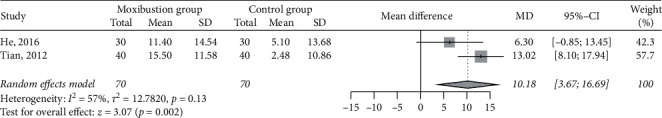
Meta-analysis of moxibustion versus nonmoxibustion treatment for quality of life.

Two RCTs (*n* = 140) [32, 40] reported data on quality of life. Both were based on the Kidney Disease Quality of Life-Short Form (KDQOL-SF) scale. Patients treated with moxibustion had a significant improvement in quality of life compared with those treated with basic treatments only (MD 10.18, 95% CI 3.67 to 16.69, *P*=0.002; I^2^ = 57%; Figure 9).

### 3.11. Subgroup Analysis

The subgroup analyses found two sources of heterogeneity in serum creatinine: patients receiving >8 weeks of moxibustion and indirect moxibustion (−58.35 versus −2.78 *μ*mol/L, interaction *P*=0.01) and direct moxibustion (−66.25 versus −6.12 *μ*mol/L, interaction *P*=0.007), respectively. There were no significant subgroup differences among the other subgroup analyses (all tests for subgroup differences: *P* > 0.05). The details of subgroup analyses are represented in Table S2 and Figures S1–S15 in the supplementary files.

### 3.12. Sensitivity Analysis

After excluding the RCTs with a high risk of bias, only the result of blood urea nitrogen had an important change (main analysis: MD −0.63 mmol/L, 95% CI −1.09 to −0.18, *p*=0.006 versus sensitivity analysis: MD −0.96 mmol/L, 95% CI −2.96 to 1.03, *p*=0.340). See the details in Table S3 in the supplementary files.

### 3.13. Publication Bias

The funnel charts of 24 hour urine protein excretion, serum creatinine, and blood urea nitrogen were symmetrical (Figures S16–S18 in the supplementary files), and the *p* values of Egger's regression test were 0.761, 0.345, and 0.562, respectively, indicating that there was no significant publication bias in these outcomes. Publication bias was not tested for the other outcomes because of insufficient sample sizes.

### 3.14. Safety Analysis

Four out of 23 RCTs [31, 34, 38, 43] reported adverse event information. One study reported [34] that one patient was burned and developed blistering on the right side of the ST36 acupoint after moxibustion, which was recovered without specific treatment. The other three RCTs reported that no adverse reactions occurred in either group.

## 4. Discussion

This systematic review included a total of 23 small RCTs, and the pooled results suggested that compared with basic treatment alone, moxibustion-assisted treatment could significantly reduce serum creatinine, blood urea nitrogen, and 24 hour urine protein excretion and improve the quality of life (assessed by only two RCTs) in patients with CKD. However, moxibustion may not help to improve eGFR, creatinine clearance, or hemoglobin.

As a metabolite of creatine phosphate excreted through the kidney, serum creatinine can sensitively reflect the progression of CKD [53]. After moxibustion therapy, both patients with mild-to-moderate CKD and patients with severe CKD had significantly reduced serum creatinine levels (−16.79 and −33.81 *μ*mol/L, respectively). The decreasing effects of moxibustion on blood urea nitrogen and 24 hour urine protein excretion were close to those of serum creatinine [54], which can be regarded as a kind of consistency verification.

The results of subgroup analysis suggested that a longer course of moxibustion (>8 weeks) and indirect moxibustion had a greater effect on reducing serum creatinine, which is consistent with our hypothesized effect direction. A lot of evidence has demonstrated that moxibustion is a therapy that requires a long-term dose to achieve the desired effect [14]. The indirect moxibustion burns moxa on top of herbs such as ginger, garlic, and aconite cakes in which the thermal stimulation becomes more even and intense and the herbs used for separation can produce additional treatment effects [55, 56]. Therefore, the subgroup hypotheses can also be explained in principle, and the credibility of the subgroup findings is increased.

After moxibustion treatment, the quality of life of patients with CKD was also improved. The tool used for evaluation was the validated KDQOL-SF scale [57], with a score ranging from 0 to 100 points. Currently, the minimum clinically important difference in KDQOL-SF has not been established, while a 10.18-points increase in KDQOL-SF should be of clinical significance for the patients. Anemia is an important factor affecting the quality of life of patients with CKD [58], but our results found that the level of hemoglobin was not improved after moxibustion. Therefore, the improvement of the quality of life by moxibustion may not be associated with the control of anemia but may be more dependent on the reduction of the level of metabolites such as serum creatinine.

The mechanisms underlying the therapeutic effects of moxibustion for CKD have yet to be clarified. Some studies found that moxa heat could dilate local renal capillaries through meridian and collateral channels [20, 59], and chronic heat stimulation may also help to alleviate the damage of renal microcirculation [60]. On the molecular biology level, the effects of moxibustion against CKD may be associated with enhancing the expression of renal podocin and nephrin mRNA and increasing the level of podocyte marker protein in kidney tissue to alleviate kidney podocyte injury [20]. Another rabbit model experiment suggested that moxibustion could inhibit the expression of connective tissue growth factors and integrin-linked kinase and upregulate the expression of bone morphogenetic protein 7 in renal tissue, resulting in a reduction in the abnormal deposition of the extracellular matrix and an inhibition of tubulointerstitial fibrosis [61].

The level of eGFR was not improved after moxibustion. Therefore, the negative eGFR result means that moxibustion may not ultimately recover the renal function. Moxibustion also did not exert a significant effect on another indicator reflecting filtration capacity and creatinine clearance, which can be considered a confirmation of the eGFR. Nevertheless, it was noted that the total sample sizes of both eGFR and creatinine clearance were small, and thus, the accuracy of the results was insufficient.

Although moxibustion is a noninvasive therapy, patients with CKD are prone to suffer from adverse events such as burns due to the decreased immunity [62]. Therefore, we hoped to elaborate the safety of moxibustion in CKD in this review. However, the majority of RCTs were short in length of follow-up and did not report the information on adverse events [63], so the safety of moxibustion cannot be sufficiently evaluated in this systematic review. In fact, it is not rare to see the reports of adverse events after moxibustion from non-CKD patients/subjects, such as burns and fever, cellulitis, and abscess after being burned, as well as allergies, cough, nausea, and vomiting caused by the moxa heat and smoke stimulations [64–68]. Therefore, we suggest that moxibustion should be performed by skilled doctors, and more attention should be paid to the distance and dose for patients with CKD.

To the best of our knowledge, this is the first systematic review focusing on the efficacy and safety of moxibustion in the treatment of CKD patients. The comprehensive literature search and strict implementation of predesigned data analysis and quality assessment protocols are the main methodological advantages in our review. However, the review still has some limitations. First, the risk of bias of the RCTs was moderate to high. Although the sensitivity analyses excluding the RCTs with a high risk of bias showed that most of the results did not change significantly, the real effect values may inevitably be biased. Especially for blood urea nitrogen, the result direction was changed after excluding RCTs with a high risk of bias. Second, although a small part of heterogeneity was explained by the subgroup analyses, the residual heterogeneity remained high in some outcomes (e.g., serum creatinine and 24 hour urine protein excretion) and the quality of evidence of these outcomes should be rated down accordingly. Third, the sample size in meta-analysis was still small in some outcomes (e.g., eGFR and quality of life); therefore, the accuracy of effect estimates was insufficient for these outcomes. Fourth, we planned to analyze the effect of moxibustion on urine albumin to creatinine ratio in the protocol, but no evidence was found.

## 5. Conclusions

The current RCT evidence shows that moxibustion, as an adjuvant therapy to basic treatments, may have effects on improving serum creatinine, urinary protein excretion, and quality of life in patients with CKD and possibly improve blood urea nitrogen. Moxibustion may not impact eGFR, creatinine clearance, or hemoglobin based on the results of the meta-analysis. The quality of evidence is weakened by the limitations including moderate-to-high risk of bias, unexplained heterogeneity, and imprecision. Well-designed, large-sample RCTs are warranted to verify the results of this review, and the long-term efficacy and safety of moxibustion for CKD also remain to be tested.

## Figures and Tables

**Table 1:** Characteristics of the included studies.

Author	No. of patients (MG/CG)	Mean age (MG/CG, year)	Details of moxibustion				Baseline renal function
			Type	Dose	Course	Acupoint	
Cheng, 2012 [26]	30/30	57.3/56.9	Ginger-separated moxibustion	3 cones/session, 7 sessions/week	1-2 weeks	CV12, CV08	Undergoing peritoneal dialysis
							SCr 650.4 ± 190.3 *μ*mol/L
Deng, 2016 [27]	30/29	42.3/43.5	Direct moxibustion	15 mins/session, 7 sessions/week	4 weeks	CV12, CV04, CV08, CV06, K11	CKD stage 2-3
							eGFR 52.4 ± 17.0 ml/min/1.73 m^2^
							SCr 141.8 ± 38.7 *μ*mol/L
He, 2016 [28]	30/30	Not reported	Direct moxibustion	30 mins/session, 14 sessions/week	2 weeks	CV12, CV04, CV08, CV06	CKD stage 4-5
Liang, 2018 [29]	32/32	42.4/41. 3	Long-snake moxibustion	5 cones/session, 0.5 session/week	3 weeks	From GV14 to GV02	CKD stage 2-3
							24 hUPE 4.98 ± 1.53 g/24h
Qiu, 2012 [30]	58/51	57.8/57.5	Direct moxibustion	1-2 cones/session, 2-3 sessions/week	6 weeks	ST36, SP06	Undergoing hemodialysis
Rong, 2017 [31]	32/32	45.7/44.9	Direct moxibustion	40 mins/session, 5 sessions/week	8 weeks	CV04, SP06	CKD stage 2-3
							24 hUPE 3.36 ± 2.54 g/24 h
Shen, 2013 [32]	28/30	52.7/58.1	Direct moxibustion	10–15 min/session, 3-4 sessions/week	3 weeks	BL20, BL23, BL28, BL22, ST36, SP06	CKD stage 2-3
							SCr 79.58 ± 11.05 *μ*mol/L
Sun, 2008 [33]	37/34	63.2/62.7	Direct moxibustion	2 cones/session, 2-3 sessions/week	12 weeks	ST36, SP06, CV04	Undergoing hemodialysis
Sun, 2017 [34]	30/30	56.8/59.1	Direct moxibustion	20 mins, 3-4 sessions/week	12 weeks	BL23, ST36, GV03, GV12	CKD stage 4-5
							SCr 357.23 ± 142.00 *μ*mol/L
Sun, 2012 [35]	51/58	57.8/57.5	Direct moxibustion	1-2 cones/session, 2-3 sessions/week	2 weeks	ST36, SP06	Undergoing hemodialysis
Tian, 2012 [36]	40/40	43.5/44.3	Direct moxibustion	20–50 mins/session, 7 sessions/week	3 weeks	ST36, CV04, CV06, BL13, BL20, BL23, GV14, GV08, GV04	CKD stage 2-3
							SCr 76.53 ± 11.55 mmol/L
Wang, 2000 [37]	28/40	47/45	Herbal cake-separated moxibustion	2 cones/session, 6 sessions/week	12 weeks	GV14, GV04, BL23, BL20, CV12, ST36, CV03	Undergoing hemodialysis
							SCr 679.38 ± 203.67 *μ*mol/L
Wang, 2013 [38]	30/30	45.2/46.4	Aconite cake-separated moxibustion	3 cones/session, 5 sessions/week	8 weeks	GV14, BL23, BL20, CV12, ST36, SP06, CV03	CKD stage 2-3
							SCr 221.35 ± 77.53 *μ*mol/L
Wang, 2017 [39]	42/40	32.3/35.5	Direct moxibustion	30 mins/session, 5 sessions/week	8 weeks	CV04	CKD stage 2-3
							eGFR 68.29 ± 37.38 ml/min/1.73 m^2^
Wang, 2018 [40]	38/38	32.3/35.5	Aconite cake-separated moxibustion	7 cones/session, 3 sessions/week	8 weeks	CV07	CKD stage 2-3
							24 hUPE 1.90 ± 1.03 g/24 h
Wen, 2018 [41]	30/30	49.5/48.9	Ginger-separated moxibustion	6–9 cones/session, 7 sessions/week	2-3 weeks	ST36, SP06, GV04, BL23, GV06	CKD stage 4-5
							24 hUPE 1.56 ± 0.35 g/24 h
Yang, 2017 [42]	30/30	22.4/22.5	Direct moxibustion	15 mins/session, 2 sessions/week	12 weeks	GV04, BL20, BL23, KI03, SP06, ST36, SP 09	CKD stage 2-3
							SCr 136.70 ± 12.52 *μ*mol/L
Zhao, 1995 [43]	33/15	52 (total)	Herbal cake-separated moxibustion	3 cones/session, 3.5 sessions/week	7 weeks	GV14, GV04, BL23, BL20, CV17, CV12, CV08, ST36	CKD stage 4-5
							SCr 687.92 ± 183.36 *μ*mol/L
Zhao, 2016 [44]	30/29	69.7/69.3	Direct moxibustion	1 cone/session, 3 sessions/week	12 weeks	LI10, ST36, SP10, BL13, HT07	Undergoing hemodialysis
							SCr 897.65 ± 329.42 mmol/L
Zhu, 2018 [45]	24/24	53.6/56.3	Direct moxibustion	30 mins/session, 7 sessions/week	8 weeks	CV12, CV08, CV04	Undergoing peritoneal dialysis
Zhuang, 2016 [46]	38/38	35.2/35.3	Long-snake moxibustion	90 mins/session, 0.25 session/week	8 weeks	From GV14 to GV04	CKD stage 2-3
							24 hUPE 4.98 ± 0.12 g/24 h
Zuo, 2015 [47]	30/30	Not reported	Herbal cake-separated moxibustion	15–18 mins/session, 5 sessions/week	12 weeks	GV04, BL23, BL20, ST36, CV04	CKD stage 2-3
							SCr 273.28 ± 102.23 *μ*mol/L
Zuo, 2018 [48]	40/40	Not reported	Herbal cake-separated moxibustion	8–10 mins/session, 5 sessions/week	12 weeks	GV04, BL23, BL20, ST36, CV04	CKD stage 2-3
							SCr 263.76 ± 89.21 *μ*mol/L

*Abbreviations*. MG = moxibustion group, CG = control group, CKD = chronic kidney diseases, eGFR = estimated glomerular filtration rate, SCr = serum creatinine, 24 hUPE = 24 hour urine protein excretion.

## Data Availability

The data used to support the findings of this study are available from the corresponding author upon request.

## Abbreviations

CKD: Chronic kidney disease
RCT: Randomized controlled trial
K/DOQI: Kidney disease outcome quality initiative
eGFR: Estimated glomerular filtration rate
MD: Mean difference
CI: Confidence interval
KDQOL-SF: Kidney disease quality of life-short.

## Supplementary Materials

Supplementary Materials Table S1: search strategy in each database. Table S2: results of subgroup analysis. Table S3: sensitivity analysis excluding trials with a high risk of bias. Figures S1–15: forest plots of subgroup analyses. Figures S16–S18: funnel plots of publication bias.
