# Supplementary material for: Moxibustion as an Adjuvant Therapy for Chronic Kidney Disease: A Systematic Review and Meta-Analysis of 23 Randomized Controlled Trials
Source: Evid Based Complement Alternat Med. 2020 Oct 28;2020:6128673. doi: 10.1155/2020/6128673 (PMC7641698; doi:10.1155/2020/6128673)
Supplement: Supplementary Materials — Table S1: search strategy in each database. Table S2: results of subgroup analysis. Table S3: sensitivity analysis excluding trials with a high risk of bias. Figures S1–15: forest plots of subgroup analyses. Figures S16–S18: funnel plots of publication bias. [file 6128673.f1.pdf]

**Table S1. Search strategy in each database**

| Database | Search strategy |
|---|---|
| **PubMed** | (renal insufficiency, chronic[mh] OR renal replacement therapy[mh] OR diabetic nephropathies[mh] OR nephropathy*[tw] OR renal function*[tw] OR kidney function*[tw] OR renal disease*[tw] OR kidney disease*[tw] OR renal failure*[tw] OR kidney failure*[tw] OR renal insufficien*[tw] OR kidney insufficien*[tw] OR albuminuri*[tw] OR microalbuminuri*[tw] OR macroalbuminuri*[tw] OR proteinuri*[tw] OR albumin excretion*[tw] OR protein excretion*[tw] OR glomerular filtration rate*[tw] OR creatinine*[tw] OR dialy*[tw]) AND (moxibustion [mh] OR moxibustion[tw] OR moxa[tw]) NOT (animals[mh] NOT humans[mh]) |
| **Embase** | (renal insufficiency, chronic/exp OR renal replacement therapy/exp OR diabetic nephropathy/exp OR nephropathy*:ti,ab OR renal function*:ti,ab OR kidney function*:ti,ab OR renal disease*:ti,ab OR kidney disease*:ti,ab OR renal failure*:ti,ab OR kidney failure*:ti,ab OR renal insufficien*:ti,ab OR kidney insufficien*:ti,ab OR albuminuri*:ti,ab OR microalbuminuri*:ti,ab OR macroalbuminuri*:ti,ab OR proteinuri*:ti,ab OR albumin excretion*:ti,ab OR protein excretion*:ti,ab OR glomerular filtration rate*:ti,ab OR creatinine*:ti,ab OR dialy*:ti,ab) AND (moxibustion/exp OR moxibustion:ti,ab OR moxa:ti,ab) |
| **The Cochrane Library** | 1. nephropathy*:ti,ab OR renal function*:ti,ab OR kidney function*:ti,ab OR renal disease*:ti,ab OR kidney disease*:ti,ab OR renal failure*:ti,ab OR kidney failure*:ti,ab OR renal insufficien*:ti,ab OR kidney insufficien*:ti,ab OR albuminuri*:ti,ab OR microalbuminuri*:ti,ab OR macroalbuminuri*:ti,ab OR proteinuri*:ti,ab OR albumin excretion*:ti,ab OR protein excretion*:ti,ab OR glomerular filtration rate*:ti,ab OR creatinine*:ti,ab OR dialy*:ti,ab<br>2. MeSH descriptor: [Renal Insufficiency, Chronic] explode all trees<br>3. MeSH descriptor: [Renal Replacement Therapy] explode all trees<br>4. MeSH descriptor: [Renal Replacement Therapy] explode all trees<br>5. #1 OR #2 OR #3 OR #4<br>6. MeSH descriptor: [Moxibustion] explode all trees<br>7. moxibustion:ti,ab OR moxa:ti,ab<br>8. #6 OR #7<br>9. #5 AND #8 (limited to “trials”) |
| **Clinicaltrials.gov** | Condition or disease: Chronic Kidney Diseases<br>Intervention/treatment: moxibustion |
| **SinoMed** | (灸法[主题词] OR 灸[全部字段]) AND (肾脏病[主题词] OR 肾炎[主题词] OR 肾病[全部字段] OR 肾炎[全部字段] OR 肾脏病[全部字段] OR 蛋白尿[全部字段] OR 肌酐[全部字段] OR 透析[全部字段] OR 血透[全部字段] OR 腹透[全部字段]) |

| | |
|---|---|
| **Chinese National Knowledge Infrastructure** | TI=灸*(肾+蛋白尿+肌酐+透析+血透+腹透) AND AB=灸*(肾病+肾炎+肾脏病+蛋白尿+肌酐+透析+血透+腹透) |
| **WanfangData** | 主题:灸*("肾病"+"肾炎"+"肾脏病"+"蛋白尿"+"肌酐"+"透析"+"血透"+"腹透") |
| **VIP** | U=灸 AND (U=肾病 OR U=肾炎 OR U=肾脏病 OR U=蛋白尿 OR U=肌酐 OR U=透析 OR U=血透 OR U=腹透) |
| **Chinese Clinical Trial Registry** | 研究疾病名称:肾病<br>干预措施:灸 |

## Table S2. Results of subgroup analysis

| Outcomes | Analysis | Subgroup | Mean difference (95% confidence intervals) | Heterogeneity | Interaction p |
|---|---|---|---|---|---|
| Serum creatinine | All patients | | -17.34 (-28.44, -6.23) | $I^2$=87%, P<0.001 | |
| | Stage of chronic kidney disease | Mild to moderate | -16.97 (-28.33, -5.52) | $I^2$=90%, P<0.001 | P=0.62 |
| | | Severe | -33.41 (-87.99, 25.17) | $I^2$=0%, P=0.90 | |
| | Type of moxibustion | Direct | -6.12 (-15.16, 2.93) | $I^2$=82%, P<0.001 | P=0.007 |
| | | Indirect | -66.25 (-108.80, -23.70) | $I^2$=71%, P=0.02 | |
| | Length of follow-up | ≤ 8 weeks | -2.78 (-9.69, 4.13) | $I^2$=60%, P=0.02 | P=0.01 |
| | | > 8 weeks | -58.35 (-102.46, -14.25) | $I^2$=84%, P<0.001 | |
| 24-hour urine protein excretion | All patients | | -0.75 (-1.07, -0.42) | $I^2$=84%, P<0.001 | |
| | Stage of chronic kidney disease | Mild to moderate | -0.75 (-1.07, -0.42) | $I^2$=84%, P<0.001 | |
| | | Severe | No studies | | |
| | Type of moxibustion | Direct | -0.82 (-1.32, -0.33) | $I^2$=89%, P<0.001 | P=0.41 |
| | | Indirect | -0.60 (-0.77, -0.43) | $I^2$=0%, P=0.96 | |
| | Length of follow-up | ≤ 8 weeks | -0.77 (-1.16, -0.38) | $I^2$=86%, P<0.001 | P=0.40 |
| | | > 8 weeks | -0.58 (-0.80, -0.36) | $I^2$=0%, P=0.96 | |
| Creatinine clearance | All patients | | 2.44 (-0.46, 5.35) | $I^2$=52%, P=0.10 | |
| | Stage of chronic kidney disease | Mild to moderate | 4.36 (-5.48, 14.21) | $I^2$=81%, P=0.02 | P=0.70 |
| | | Severe | 2.34 (-0.59, 5.26) | $I^2$=25%, P=0.26 | |
| | Type of moxibustion | Direct | 3.87 (0.73, 7.01) | $I^2$=0%, P=0.56 | P=0.49 |
| | | Indirect | 1.96 (-2.53, 6.45) | $I^2$=65%, P=0.06 | |
| | Length of follow-up | ≤ 8 weeks | 3.42 (-0.12, 6.97) | $I^2$=50%, P=0.11 | P=0.15 |
| | | > 8 weeks | -0.01 (-3.09, 3.07) | Not applicable | |
| Blood urea nitrogen | All patients | | -0.63 (-1.09, -0.18) | $I^2$=37%, P=0.006 | |
| | Stage of chronic kidney disease | Mild to moderate | -0.67 (-1.13, -0.20) | $I^2$=40%, P=0.10 | P=0.34 |
| | | Severe | -0.91 (-2.27, 4.09) | Not applicable | |
| | Type of moxibustion | Direct | -0.62 (-0.80, -0.43) | $I^2$=0%, P=0.54 | P=0.31 |
| | | Indirect | -1.70 (-3.79, 0.40) | $I^2$=70%, P=0.02 | |
| | Length of follow-up | ≤ 8 weeks | -0.45 (-0.96, 0.06) | $I^2$=32%, P=0.21 | P=0.19 |
| | | > 8 weeks | -1.41 (-2.73, -0.08) | $I^2$=48%, P=0.10 | |

| | | | | | |
|---|---|---|---|---|---|
| Hemoglobin | All patients | | -0.41 (-3.19, 2.36) | $I^2$=0%, P=0.77 | |
| | Stage of chronic kidney disease | Mild to moderate | No studies | | |
| | | Severe | -0.41 (-3.19, 2.36) | $I^2$=0%, P=0.77 | |
| | Type of moxibustion | Direct | -1.06 (-4.15, 2.03) | $I^2$=0%, P=0.82 | P=0.35 |
| | | Indirect | 2.30 (-4.02, 8.62) | Not applicable | |
| | Length of follow-up | ≤ 8 weeks | -0.18 (-3.67, 3.30) | $I^2$=0%, P=0.63 | P=0.83 |
| | | > 8 weeks | -0.81 (-5.41, 3.78) | $I^2$=0%, P=0.35 | |

## Table S3. Sensitivity analysis excluding trials with a high risk of bias

| **Outcome** | **Analysis** | **No. of patients** | | **Mean difference (95% confidence intervals)** | **Heterogeneity** |
|---|---|---|---|---|---|
| | | **Moxibustion** | **Control** | | |
| Serum creatinine | Main analysis | 423 | 415 | -17.34 (-28.44, -6.23) | $I^2$=87%, P=0.002 |
| | *Excluding trials with high risk of bias* | 265 | 243 | -32.09 (-55.46, -8.72) | $I^2$=86%, P=0.007 |
| Estimated glomerular filtration rate | Main analysis | 72 | 69 | 1.93 (-14.41, 18.28) | $I^2$=71%, P=0.82 |
| | *Excluding trials with high risk of bias* | 72 | 69 | 1.93 (-14.41, 18.28) | $I^2$=71%, P=0.82 |
| 24-hour urine protein excretion | Main analysis | 330 | 329 | -0.75 (-1.07, -0.42) | $I^2$=84%, P<0.001 |
| | *Excluding trials with high risk of bias* | 170 | 167 | -0.56 (-0.72, -0.40) | $I^2$=0%, P<0.001 |
| Creatinine clearance | Main analysis | 144 | 144 | 2.44 (-0.46, 5.35) | $I^2$=52%, P=0.10 |
| | *Excluding trials with high risk of bias* | 84 | 84 | 3.84 (-1.03, 8.72) | $I^2$=72%, P=0.12 |
| Blood urea nitrogen | Main analysis | 335 | 327 | -0.63 (-1.09, -0.18) | $I^2$=37%, P=0.006 |
| | *Excluding trials with high risk of bias* | 128 | 140 | -0.96 (-2.96, 1.03) | $I^2$=69%, P=0.34 |
| Hemoglobin | Main analysis | 207 | 194 | -0.41 (-3.19, 2.36) | $I^2$=0%, P=0.77 |
| | *Excluding trials with high risk of bias* | 147 | 134 | -1.17 (-4.84, 2.50) | $I^2$=0%, P=0.53 |

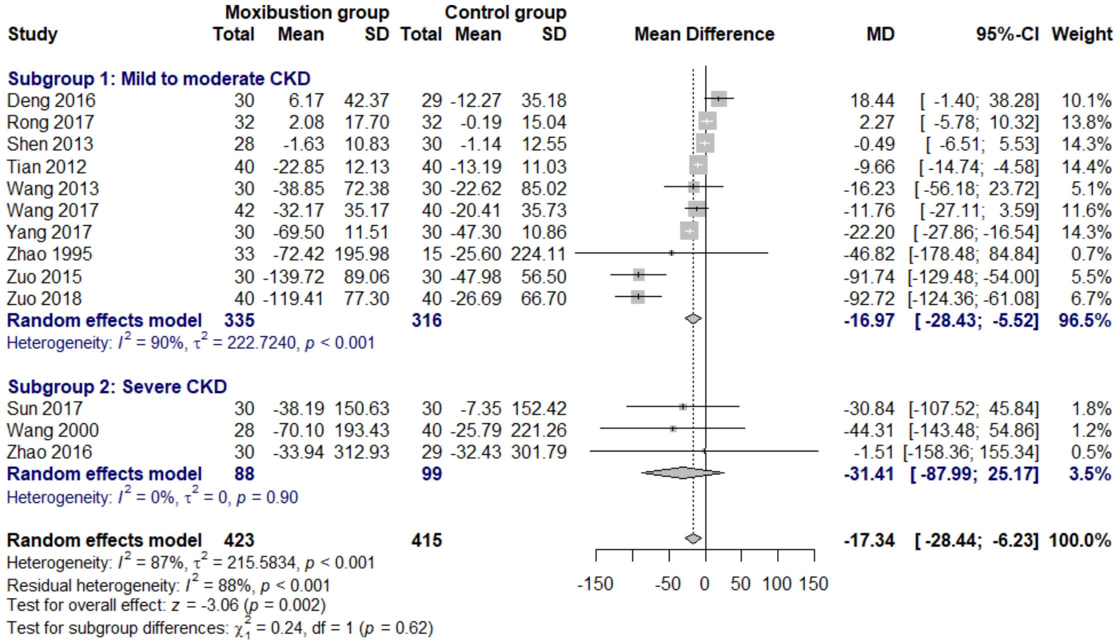


Figure S1. Subgroup analysis stratified by different stage of moxibustion for changes in serum creatinine (μmol/L)

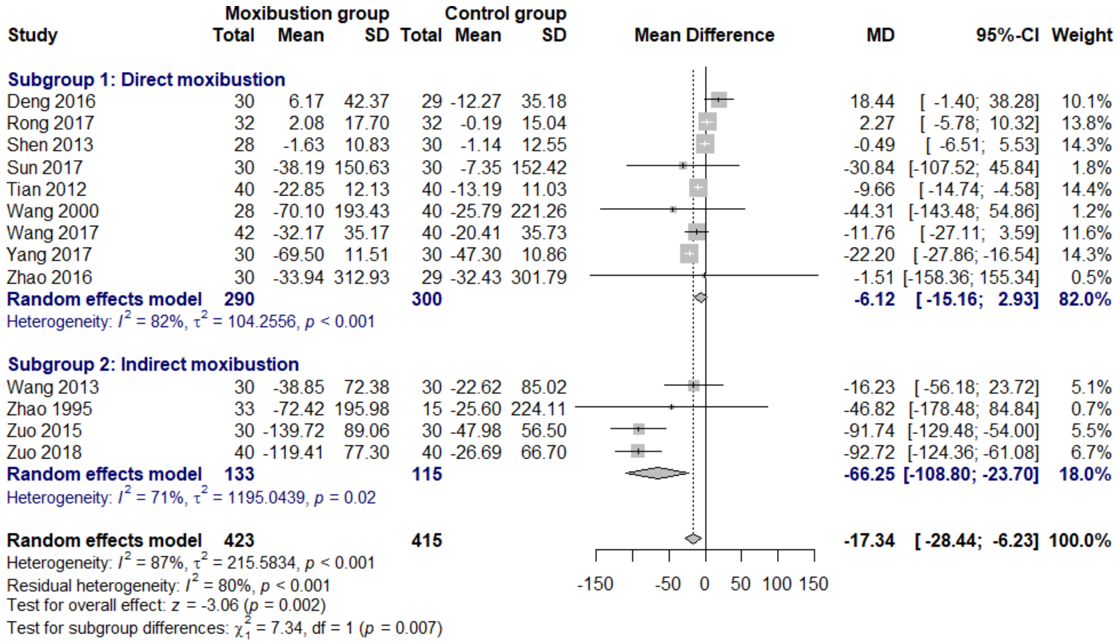


Figure S2. Subgroup analysis stratified by different type of moxibustion for changes in serum creatinine (μmol/L)

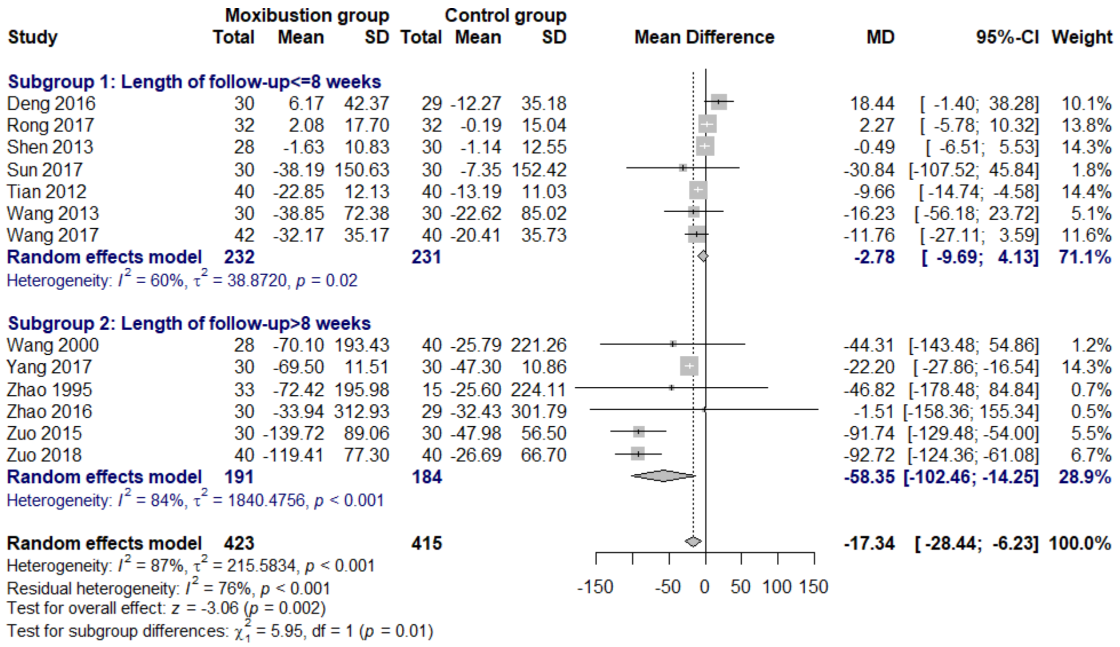


Figure S3. Subgroup analysis stratified by different follow-up of moxibustion for changes in serum creatinine (μmol/L)

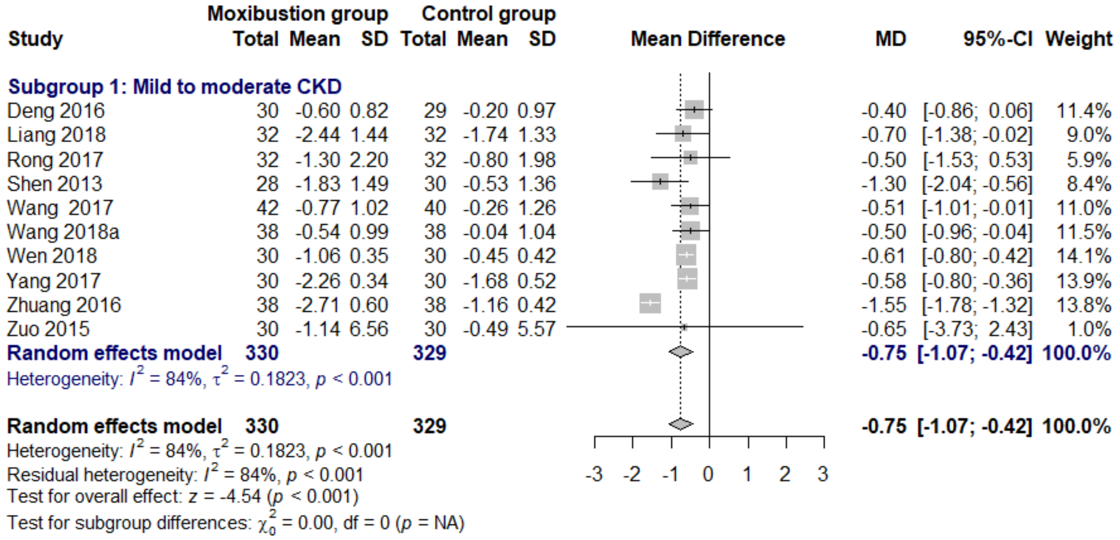


Figure S4. Subgroup analysis stratified by different stage of moxibustion for changes in 24-hour urine protein excretion (g/h)

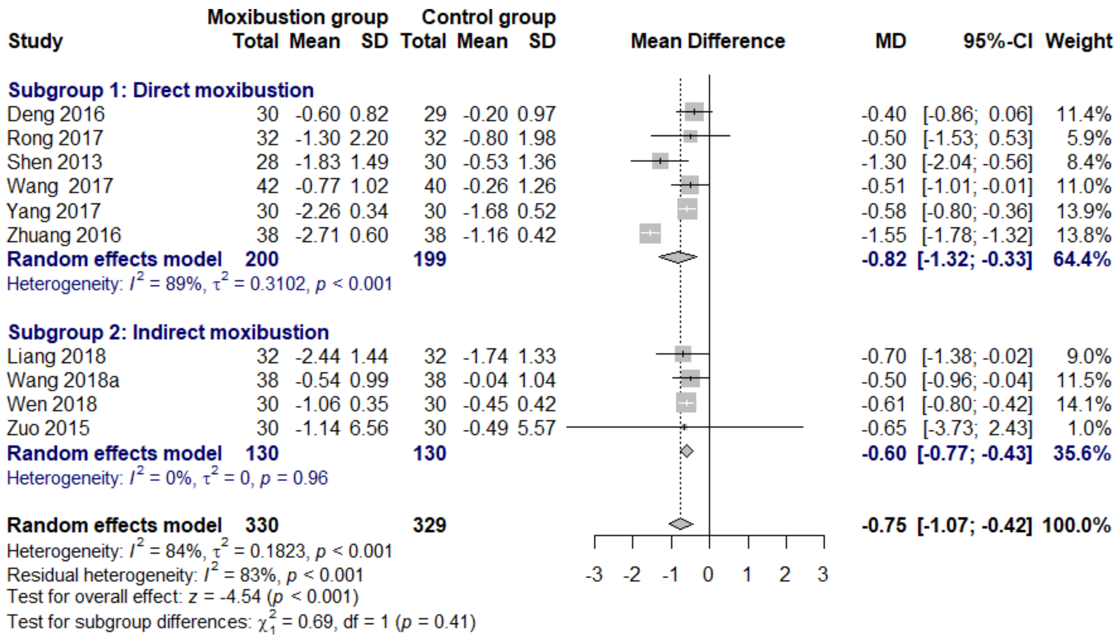


Figure S5. Subgroup analysis stratified by different type of moxibustion for changes in 24-hour urine protein excretion (g/h)

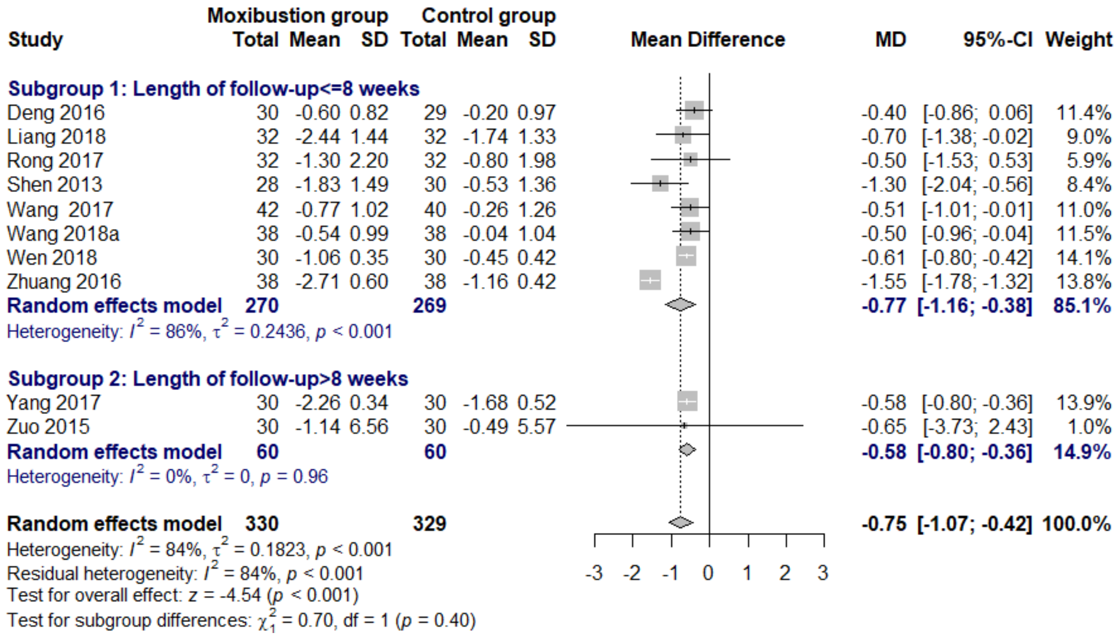


Figure S6. Subgroup analysis stratified by different follow-up of moxibustion for changes in 24-hour urine protein excretion (g/h)

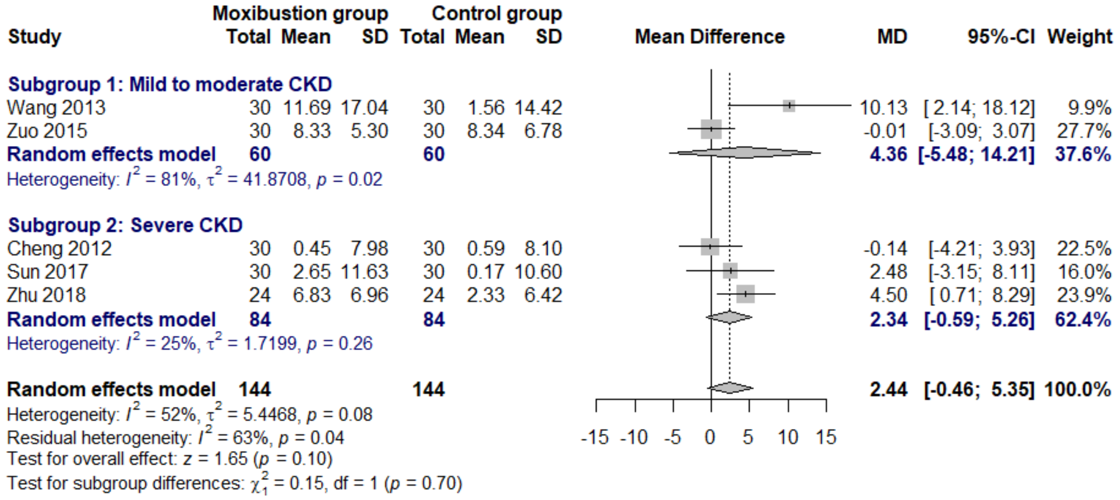


Figure S7. Subgroup analysis stratified by different stage of moxibustion for changes in creatinine clearance (mL/min)

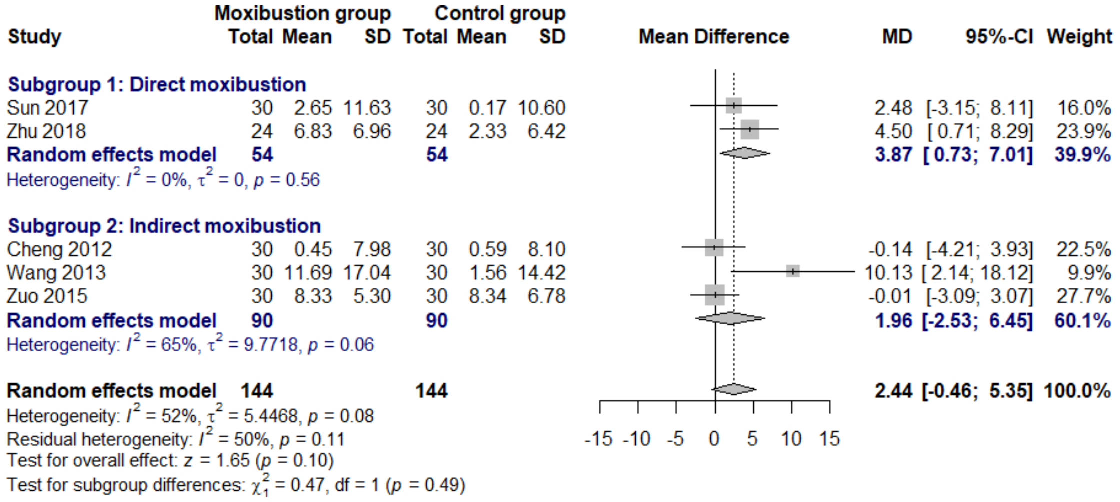


Figure S8. Subgroup analysis stratified by different type of moxibustion for changes in creatinine clearance (mL/min)

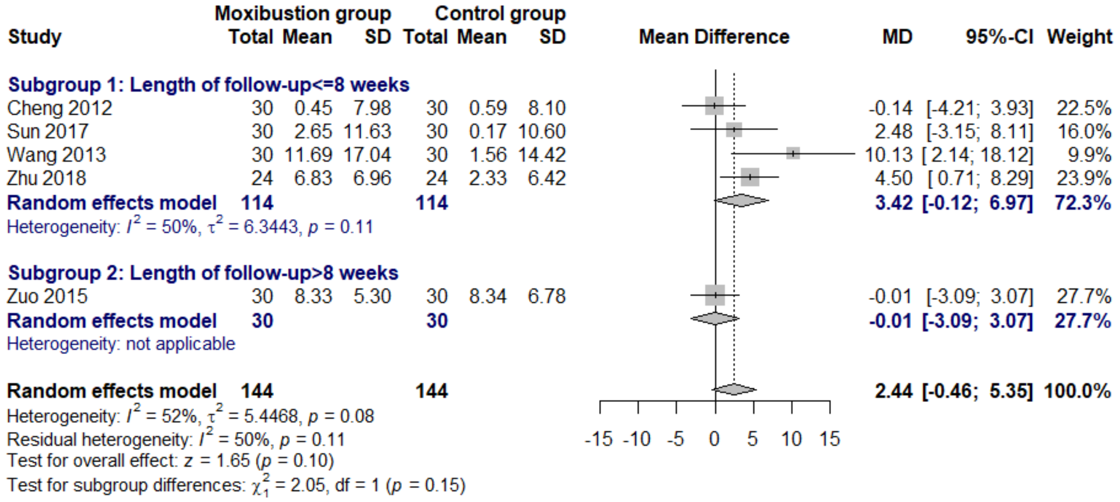


Figure S9. Subgroup analysis stratified by different follow-up of moxibustion for changes in creatinine clearance (mL/min)

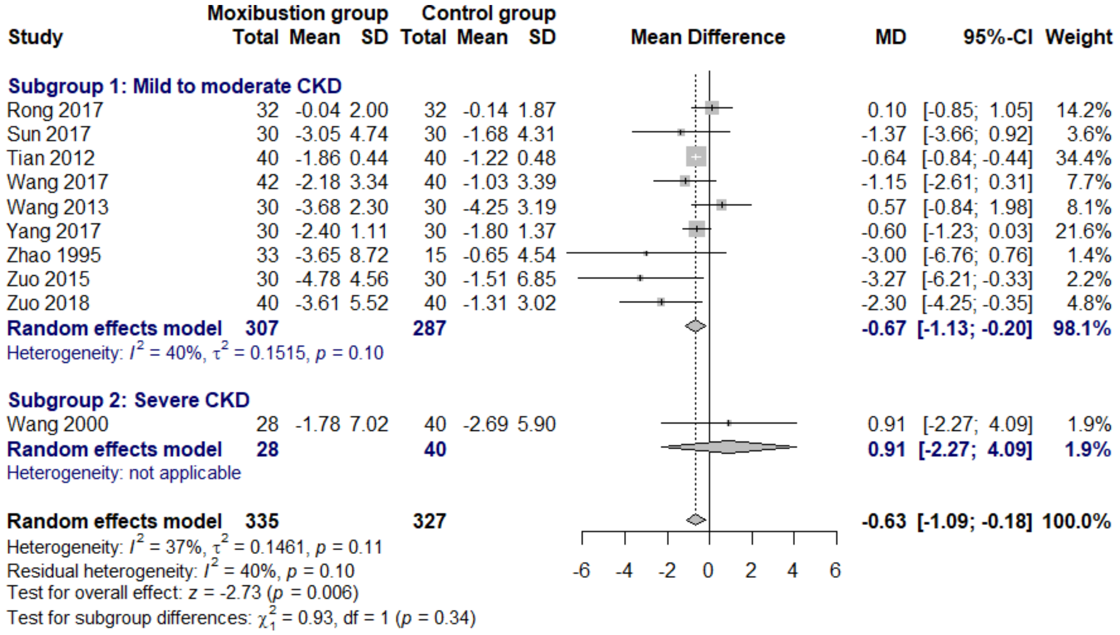


Figure S10. Subgroup analysis stratified by different stage of moxibustion for changes in blood urea nitrogen (mmol/L)

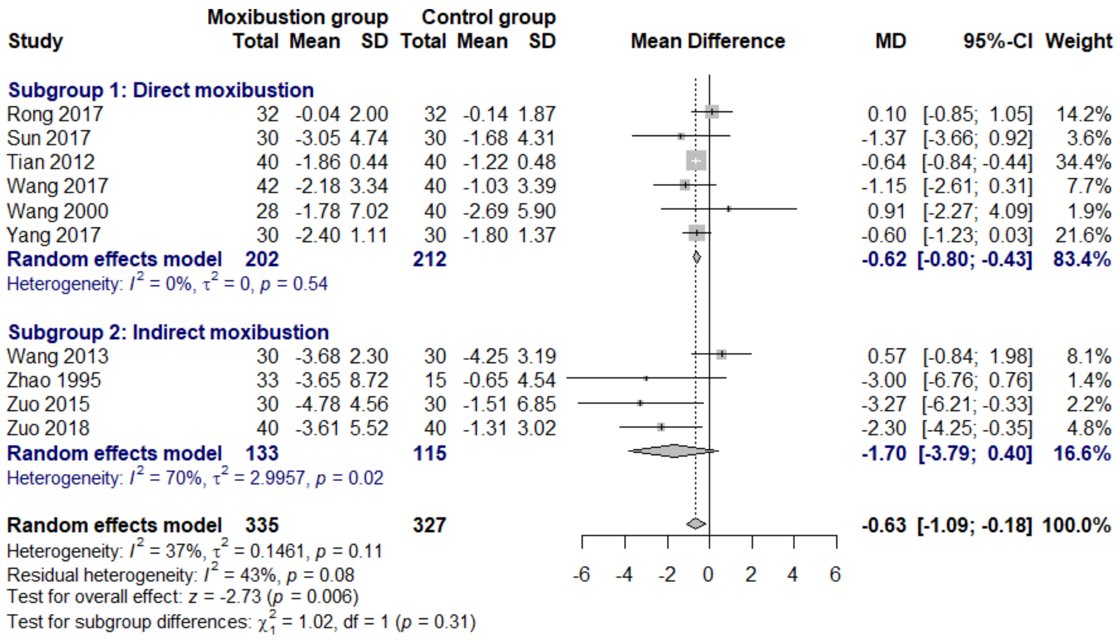


Figure S11. Subgroup analysis stratified by different type of moxibustion for changes in blood urea nitrogen (mmol/L)

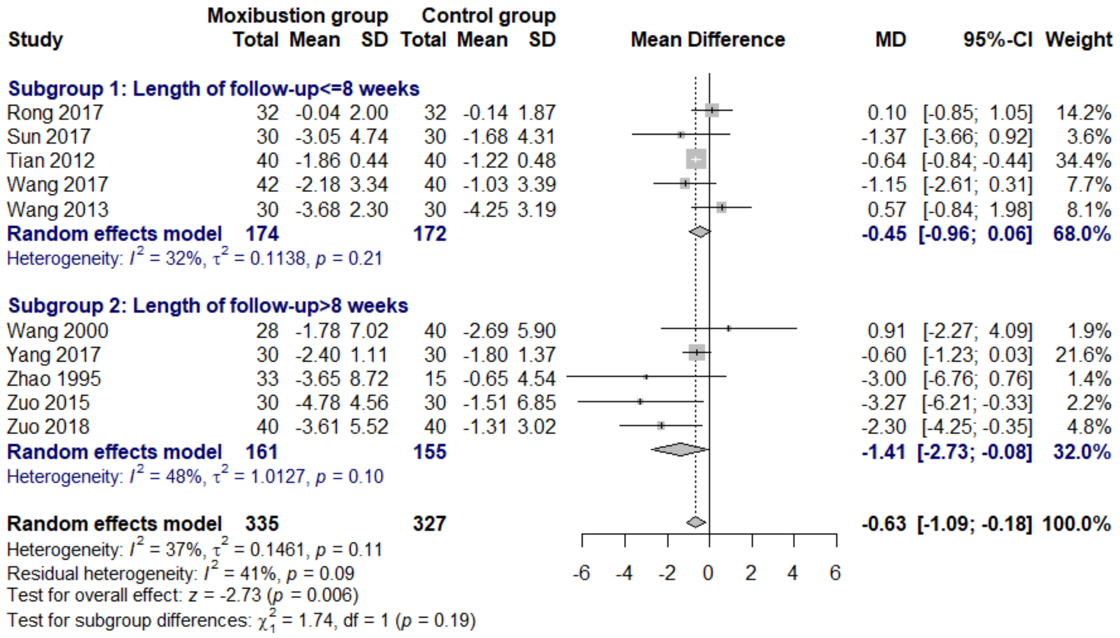


Figure S12. Subgroup analysis stratified by different follow-up of moxibustion for changes in blood urea nitrogen (mmol/L)

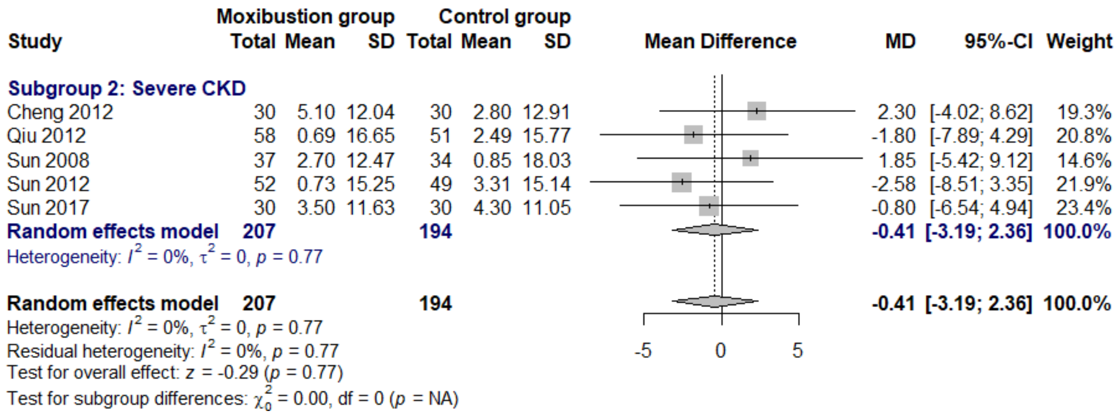


Figure S13. Subgroup analysis stratified by different stage of moxibustion for changes in hemoglobin (g/L)

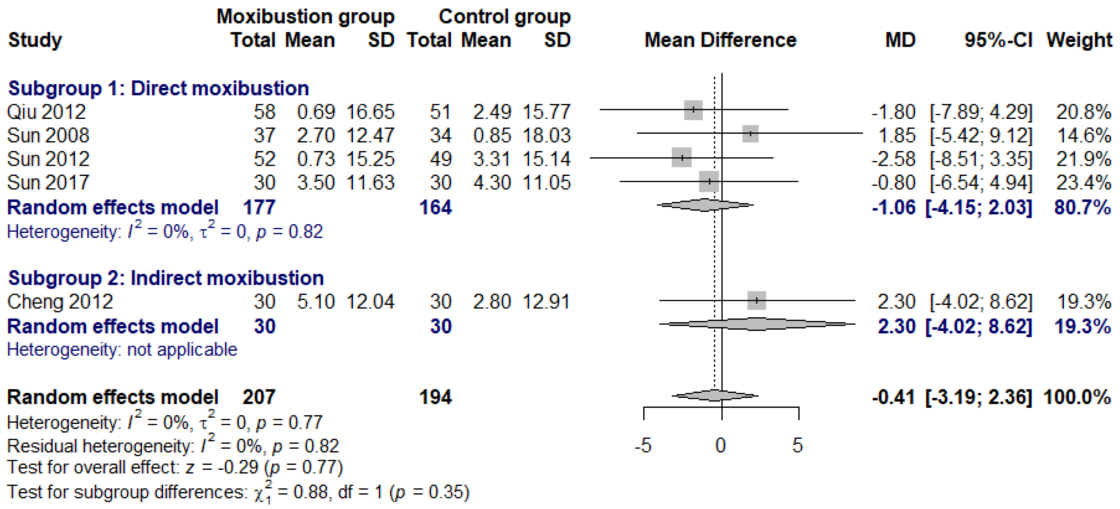


Figure S14. Subgroup analysis stratified by different type of moxibustion for changes in hemoglobin (g/L)

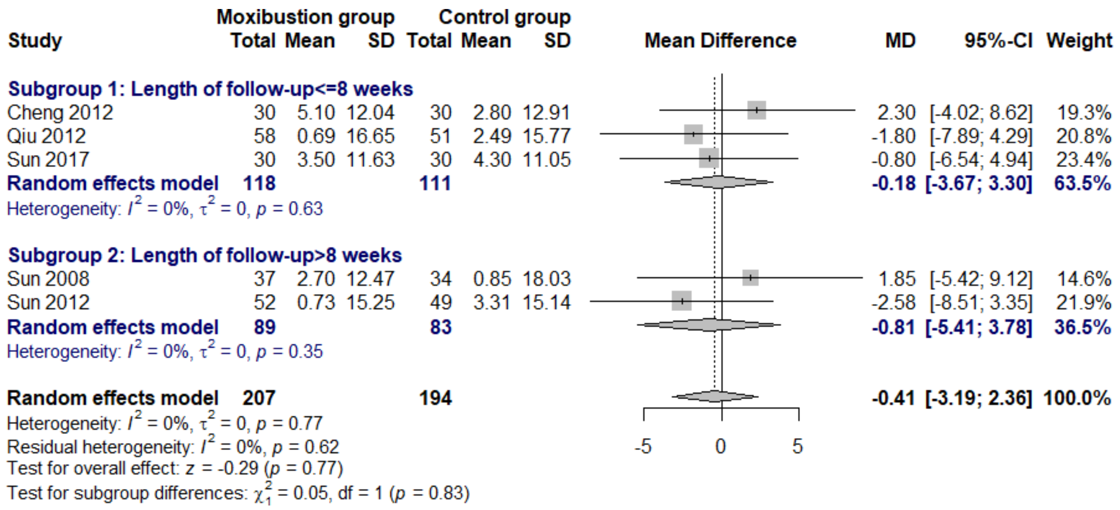


Figure S15. Subgroup analysis stratified by different follow-up of moxibustion for changes in hemoglobin (g/L)

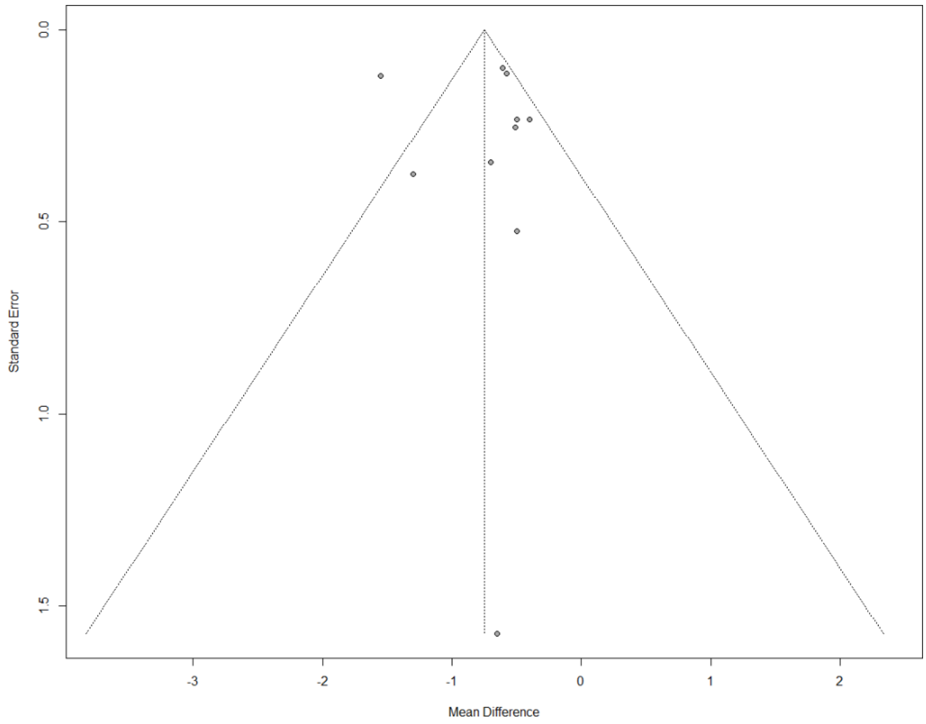


Figure S16. Funnel plot of 24-hour urine protein excretion

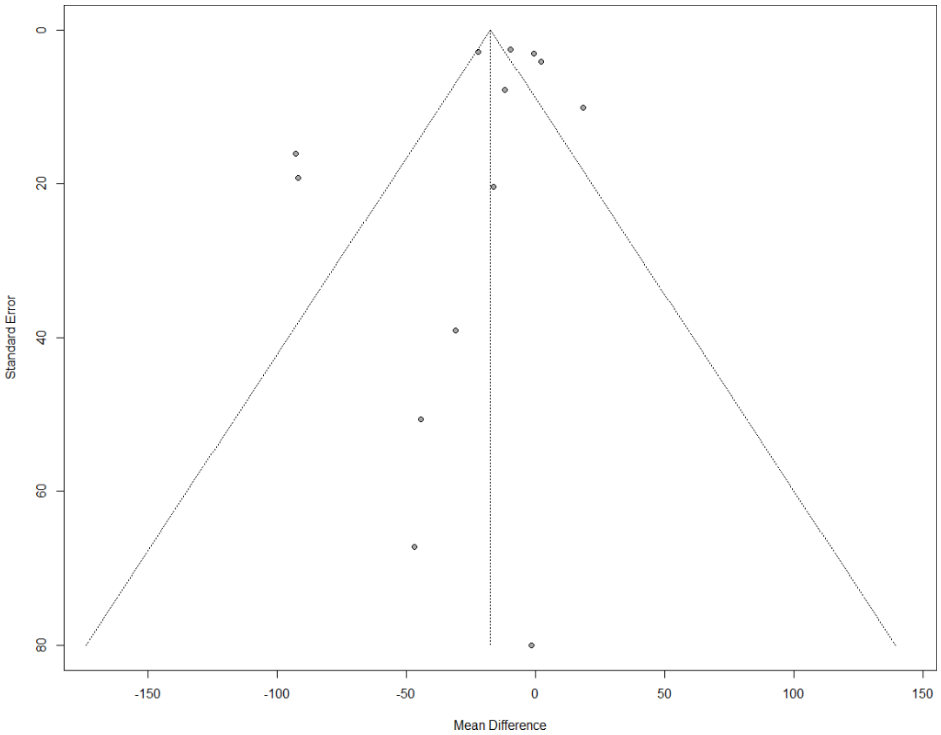


Figure S17. Funnel plot of serum creatinine

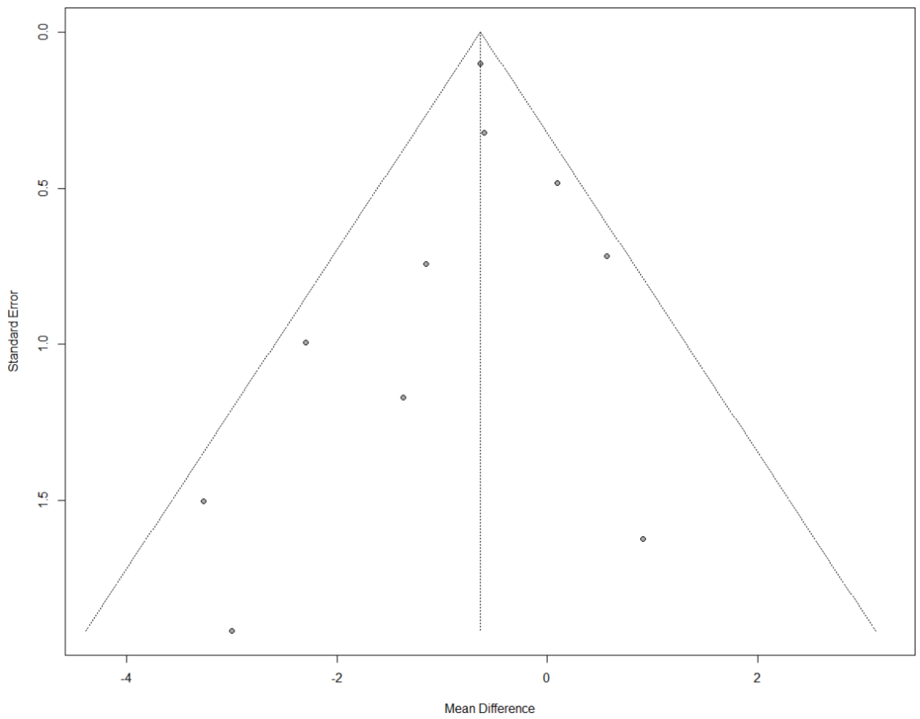


Figure S18. Funnel plot of blood urea nitrogen
